# Unfolded Protein Response and Cancer

**DOI:** 10.15190/d.2014.2

**Published:** 2014-03-14

**Authors:** Lihua Wu, Mary Chou, Shudong Zhu

**Affiliations:** School of Life Sciences, State Key Laboratory of Medical Genetics, Central South University, Changsha, China; Advanced Orthomolecular Research Inc., Calgary, Canada

**Keywords:** Unfolded Protein Response (UPR), ER stress, Cancer, PERK, IRE1a

## Abstract

Physiological stresses, such as hypoxia and oxidative stress, induce protein misfolding in the endoplasmic reticulum (ER). If proteasome degradation fails to remove the misfolded proteins, these proteins accumulate in the ER, triggering the unfolded protein response (UPR). UPR involves a series of responses, such as the suppression of global protein synthesis and the select expression of a set of proteins to reduce ER stress and restore the homeostasis of ER. In different stages of tumor development, hypoxia occurs and UPR is initiated. The roles of UPR in cancer development are complex, involving angiogenesis, cell survival and proliferation. The current knowledge of the molecular mechanisms involved in UPR, particularly its role in the development of cancer, is discussed.

## SUMMARY

1. *Introduction*
2. *Initiation of ER Stress*
3. *IRE1* *a*
4. ** *PERK*
5. *ATF6*
6. *Conclusions*

## 1. Introduction

Proteins are synthesized in the cytosolic ribosomes and in the endoplasmic reticulum (ER). After being synthesized in ER, proteins are post-translationally modified and properly folded into functional conformations before being delivered to their designated subcellular organelles. Physiological stresses such as hypoxia and oxidative stress cause a failure in proper cysteine oxidation and subsequent formation of the disulfide bonds, a critical post-translational modification, and therefore results in misfolded proteins. However, the ER contains a protein quality control system that utilizes ubiquitin-proteasome degradation to timely remove misfolded proteins. If this system fails to perform its full function, misfolded proteins will accumulate in the ER. This ER stress triggers the unfolded protein response (UPR). UPR involves a series of responses, such as suppression of global protein synthesis to reduce the burden of proliferating cells, that require a sufficient amount of properly folded proteins, and to promote synthesis of molecular chaperones to allow restoration of proper protein folding. UPR is involved in different pathological dysfunctions including cancer, neurodegeneration, inflammation, and metabolism disorders.

In stages of the development of cancer, hypoxia occurs and UPR is initiated. While the mild ER stress often leads to responses that promote tumor cell survival, extreme ER stress leads to death of cancer cells due to the insufficient proofreading ability to correct the overwhelming protein misfolding. We review the current progress of the molecular mechanisms involved in UPR, particularly their roles in the development of cancer.

## 2. Initiation of ER Stress 

ER stress can be triggered in various ways. When the growth of a tumor exceeds the supply of sufficient oxygen, hypoxia in the tumor microenvironments results in insufficient disulfide bond formation required for proper protein folding, generating protein misfolding and hence ER stress^1^. At low glucose levels, cancer cells tend to enhance aerobic glycolysis (Warburg effect), leading to more production of lactic acid, changing the microenvironment pH, resulting in ER stress. An insufficient supply of amino acids also induces ER stress. Mitochondrial malfunction may also result in reactive oxygen species (ROS) and therefore oxidative stress, which may lead to excessive oxidative modifications to proteins and hence ER stress.

Under normal conditions, Grp78 (Bip, HspA5) protein binds transmembrane receptors of the ER, i.e., inositol requiring enzyme (IRE1α), PKR-like ER kinase (PERK) and activating transcription factor 6 (ATF6), suppressing the corresponding activities of each receptor enzyme. However, Grp78 has a high affinity for unfolded or misfolded proteins, thus under ER stress, the misfolded proteins in the ER lumen will bind to Grp78, dissociating the three transmembrane ER receptors from Grp78, resulting in the activation of the receptors. As ER stress sensors, these activated receptors will initiate a series of reactions in the cytosol and nucleus, called the unfolded protein response, which serves to correct protein misfolding and reduce ER stress as a feedback mechanism. If UPR is insufficient to correct the extent of protein misfolding, damage to the cells will occur and cell death ensues.

## 3. IRE1α

Upon dissociation from GRP78, IRE1α dimerizes and autophosphorylates, activating its kinase activity and RNase activity on its cytosolic domain^2^. IRE1α then splices and joins XBP1 mRNA resulting in a processed XBP1 mRNA that translates to the transcription factor XBP1s (spliced XBP1)^3^. XBP1s then switches on expression of a series of target genes that aim to restore the homeostasis of the ER. Most of these genes have functions involving protein folding or ER-associated protein degradation (ERAD)^4^. In addition to controlling gene expression upon ER stress, IRE1α may also assemble into the IRE1α complex to fine-tune the unfolded protein response with other adaptors and regulators^5^.

IRE1 activity has been linked to the promotion of cell survival after ER stress^6^. However, pro-survival molecules targeted by IRE1 or XBP1 have not been identified. In BaF3 cells, XBP1 knockdown induced apoptosis, while overexpression of XBP1s protected cells from apoptosis induced by IL-3 depletion^7^.

On the other hand, while IRE1α plays a predominant role to promote cell survival, there is evidence to suggest that IRE1α sometimes might also play a pro-apoptotic role in ER stress in cells: over-expression of IRE1α may trigger CHOP expression in addition to the activation of GRP78 genes, the effect of which is further proved by over-expression of a dominant-negative form of IRE1α and the over-expression of murine IRE1α^8^. Supporting this notion is that TNF receptor associated factor 2 (TRAF2) is recruited to IRE1α, linking IRE1α to the pro-apoptotic pathway of TRAF2-ASK1-JNK^9^. JNK has been shown to regulate activity of Bcl-2 family members. For example, JNK phosphorylates Bcl-2/Bcl-xL to suppress their anti-apoptotic activity as well as phosphorylates Bid/Bim, transcriptional targets of FOXO, to increase their pro-apoptotic activity^10-12^. In addition to TRAF2-ASK1-JNK signaling, IRE1α may also promote JNK signaling by increasing levels of TNFα. This is done by TRAF2 recruiting IKK to the IRE1α complex. Following IKK activation and degradation of phosphorylated IκB, NF-κB induces the expression of TNFα, which is likely to promote JNK induced apoptosis^13^. However, the interactions of IRE1α with other components and its potential role in apoptosis is largely not understood.

Besides its effect on XBP1, IRE1α may also selectively degrades a group of mRNAs that usually encode secretory proteins involved in protein folding within ER, serving to reduce the ER stress to promote cell survival, a process called regulated IRE1-dependent decay (RIDD) of mRNA^14^. A conserved nucleotide sequence may be responsible for the selectivity of IRE1α RNase activity for them^14^. Recently, IRE1α has also been reported to increase caspase-2 expression by cleaving selective premature microRNAs^15^. RIDD activation is a relatively new discovery and its targets and the mechanisms of regulation are yet to be discovered. A kinase inhibitor experiment appears to suggest that the kinase domain of the IRE1 activity might be responsible for its anti-RIDD, apoptotic role, in contrast to its RNase activity to promote XBP1-dependant cell survival^16^.

Bax Inhibitor 1 (BI-1) negatively regulates the binding of IRE1α to BAX and BAK, which would otherwise bind IRE1α at its cytoplasmic domains resulting in increased XBP1s and JNK phosphorylation^17^. BI-1 is normally ubiquitinated by bi-functional apoptosis regulator (BAR) leading to its degradation.

Bim, PUMA and Hsp72 are also able to bind IRE1α and stimulate its RNase activity, leading to increased XBP1 splicing and cell survival under ER stress^18,19^. However, the direct apoptotic role of some of these molecules (e.g., BIM) and their pro-survival role via IRE1a requires switches to fine tune the signaling under each condition. A transgenic mouse model shows XBP-1 drives multiple myeloma pathogenesis^20^. An IRE1α endonuclease inhibitor has been identified and displays cytotoxic activity against human multiple myeloma, suggesting IRE1α may be a therapeutic cancer target^21^. Furthermore, the deficiency *in *IRE1α and**XBP1 led to reduced blood vessel formation^22,23^.

## 4. PERK

**Figure 1:**
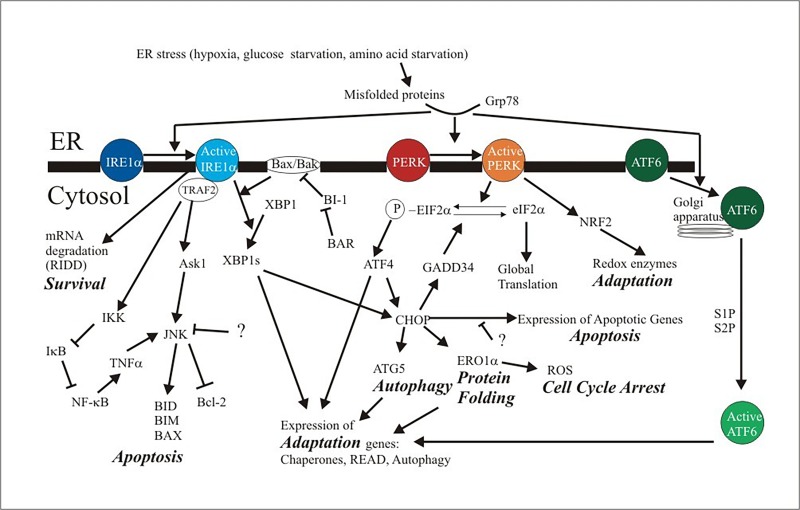
UPR signaling ER stress triggers dissociation of IRE1α, PERK and ATF6 from GRP78, activating the 3 ER stress sensors. IRE1α activates transcription factor XBP1, leading to the expression of a series of target genes that aim to restore the homeostasis of ER. Additionally, IRE1α performs RIDD to promote cell survival, and under certain situations, it might also promote apoptosis via the TRAF2/ASK1/JNK pathway and the CHOP pathway. Activated PERK disables eIF2α and suppresses global protein synthesis, and selectively promotes ATF4 to upregulate the expression of genes involved in redox, amino acid metabolism, and protein folding. ATF4 may also upregulate CHOP to induce apoptosis. Furthermore, CHOP induces expression of ERO1α to promote disulfide bond formation while generating ROS; CHOP also upregulates GADD34 to dephosphorylate eIF2α, as a feedback control to recover protein synthesis. Stress-induced ATF6 translocates to the Golgi to be processed and becomes an active transcription factor and mainly induces cytoprotective responses. The molecular mechanisms to control apoptosis while promoting cell survival have not been identified.

Under stress conditions such as amino acid starvation, UPR is initiated in cells^24^. PERK is a transmembrane protein of the ER with an ER luminal domain to sense ER stress and a cytosolic kinase domain to transduce the signal to the cytosol. Upon dissociation from GRP78, PERK dimerizes and autophosphorylates, activating its kinase domain, which then phosphorylates eukaryotic translation initiator factor 2α (eIF2α). This disables eIF2α and suppresses global protein synthesis^2,24^ (**Figure 1**), resulting in the end of cyclin D1 translation and subsequent cell cycle arrest^25^. This blockade in translation and temporary reduction in cell proliferation allows cells a chance to reduce the ER stress by reducing the amount of misfolded proteins to be synthesized, thus increasing the chance of cell survival.

However, phosphorylated eIF2α allows the selective translation of mRNAs that contains particular regulatory sequences in their 5′ UTR in the open reading frame, such as mRNA of activating transcription factor 4 (ATF4). ATF4 upregulates the expression of proteins involved in protein folding, redox, and amino acid metabolism. ATF4 also regulates the expression of proteins directly associated with apoptosis, such as the transcription factor C/EBP-homologous protein (CHOP) (GADD153), which is a key regulator of ER stress induced apoptosis, up-regulating expression BIM and down-regulating BCL-2, etc. CHOP induces the expression of ERO1α, which promotes the formation of disulfide bonds while generating ROS^26^. ATF4 also directly activates CHOP to regulate expression of growth arrest and DNA damage-inducible 34 (GADD34), which is capable of dephosphorylating eIF2α via recruiting protein phosphatase PP1C, serving as a feedback control to recover protein synthesis^26,27^. The feedback control suggests that while appropriate or light ER stress might stimulate cell survival, extreme or strong ER stress may lead to cell death.

ATF4 and CHOP have also been shown to activate the expression of microtubule-associated protein 1 light chain 3beta (MAP1LC3B) and autophagy-related gene 5 (ATG5) respectively, proteins crucial for autophagy^28^. Depending on the situations, autophagy has been shown to either enhance cell survival or promote non-apoptotic cell death^29^.

Besides eIF2α, PERK also phosphorylates nuclear factor erythroid 2-related factor-2 (NRF2), which upregulates expression of antioxidative enzymes^30^, promoting cell survival. Recently, it was also reported that the PERK-ATF4 pathway facilitates the activation ATF6 and its target genes during ER stress^31^.

ER stress has been shown to be activated in hypoxic areas of tumors, and disabling PERK by mutagenesis or a dominant-negative PERK, and disabling eIF2a by mutagenesis all lead to apoptosis under hypoxia, leading to smaller tumors and increased apoptosis, implicating the PERK pathway in promoting tumor formation^32^.

In a mammary carcinoma model, PERK was found to promote cancer cell proliferation and tumor growth by limiting oxidative DNA damage and associated cell cycle arrest^33^. This effect of PERK on cancer cell proliferation has also been observed in insulinomas induced by expression of SV40 large T-antigen, where PERK-deficient tumors are associated with reduced tumor growth. On the other hand, the same experiment also found a dramatic reduction in tumor vascularity in PERK-deficient mice. Similar observations were made in a xenograft model where PERK-deficient colorectal carcinomas were poorly vascularized, and eIF2α and ATF4 have been suggested to contribute to this effect^32^.

However, the roles of PERK in cancer cell proliferation varies in different reports. In comparison to the positive effect of PERK on cancer cell proliferation observed in insulinomas induced by expression of SV40 large T-antigen, pharmacologically-activated PERK induced squamous cell carcinoma cell growth arrest in vitro and suppressed tumor growth in vivo^34^. It is possible that Nrf2 and ROS plays an important role in the pro-proliferation effect of PERK observed in insulinoma, and reduced cyclin D1 expression plays an important role in the anti-proliferation effect of PERK observed in squamous cell carcinoma cell growth. Therefore, while the effect of PERK on tumor angiogenesis is comparatively clear, the effects of PERK on tumor growth could vary, depending on the cellular context, microenvironment and stimulus/treatment.

Calreticulin, an ER luminal protein traditionally regarded as a calcium-buffering chaperone of the endoplasmic reticulum^35^, could be expressed on the tumor cell surface during chemotherapy to induce dendritic cell-mediated phagocytosis of tumor cells, providing a new immunogenic chemotherapy for cancers. However, PERK activation to initiate eIF2/caspase 8/BAP31/BAX/BAK signaling is required for the calreticulin translocation, and inhibiting the GADD34 and PP1 complex involved in eIF2α dephosphorylation enhanced the surface exposure of calreticulin^36^.

## 5. ATF6

The ATF6 pathway in cancer is the least investigated pathway of UPR. After dissociation from GRP78 following ER stress, ATF6 translocates to the Golgi to be processed and it becomes an active transcription factor^37^. Unlike the sometimes paradoxical roles of PERK and IRE1 in inducing cell survival, ATF6 mainly induces cytoprotective responses, including the expression of genes encoding proteins that facilitate folding and the ERAD pathway, etc.^38^. ATF6 promotes survival of dormant tumor cells through the up-regulation of Rheb and activation of mTOR signaling^39^.

## 6. Conclusion

UPR signaling consists of three pathways: IRE1a, PERK, and ATF6. These pathways regulate many cellular processes including protein folding and maturation, cell survival and apoptosis, tumor growth and angiogenesis, depending on the cellular context, microenvironment and stimuli. The exact, sometimes opposing roles of these signaling molecules in cancer await further exploration.

Most recently, a lot of important progress has been made involving UPR and cancer. SirT3 has been reported as a novel key coordinator of UPR and serves as a mechanism of adaptation through orchestrating antioxidant machinery and mitophagy, implying its contrasting dual roles in cancer development^40^. A series of microRNAs have been reported to be induced in UPR to conduct cytoprotective effects or to attenuate cytoprotective effects^41,42^. A UPR element SEL1L has been recently connected to the cytotoxic effects of cancer stem cells^43^. These new discoveries are still among early efforts aiming at establishing the roles of UPR in cancer, which is largely unknown but the significance of which is beginning to be realized. Therapeutic strategies are expected to be built in the future based on a better understanding of specific roles of UPR components in various cancers. Inhibitors targeting ER stress components (e.g., ERAD inhibitor Eeyarestatin) have already revealed great potential in increasing death of cancer cells^44^.

- **Physiological stresses induce protein misfolding in the endoplasmic reticulum (ER). If proteasome degradation fails to remove misfolded proteins, these accumulate in the ER, triggering the unfolded protein response (UPR).**
- **UPR involves a series of responses, and plays** **potentially important roles in the development of cancer**
